# Editorial: Mitochondria at the Crossroads of Immunity and Inflammatory Tissue Damage

**DOI:** 10.3389/fimmu.2021.810787

**Published:** 2021-11-30

**Authors:** João Paulo Silva Nunes, Pedro M. Moraes-Vieira, Christophe Chevillard, Edecio Cunha-Neto

**Affiliations:** ^1^ Laboratory of Immunology, Heart Institute (InCor), School of Medicine, University of São Paulo, São Paulo, Brazil; ^2^ Division of Clinical Immunology and Allergy, School of Medicine, University of São Paulo, São Paulo, Brazil; ^3^ Institute for Investigation in Immunology (iii), Instituto Nacional de Ciência e Tecnologia (INCT), São Paulo, Brazil; ^4^ INSERM, UMR_1090, Aix Marseille Université, TAGC Theories and Approaches of Genomic Complexity, Institut MarMaRa, Marseille, France; ^5^ Department of Genetics, Evolution, Microbiology and Immunology, Institute of Biology, Experimental Medicine Research Cluster (EMRC), and Obesity and Comorbidities Research Center (OCRC), University of Campinas, Campinas, Brazil

**Keywords:** tissue damage, mitochondria, immunity, inflammation, Chagas

Once activated, immune cells significantly increase their energy metabolism demand through the central metabolic pathways of glycolysis and respiration. Different immune cell subsets depend on distinct energy metabolism pathways. In addition to energy production, upon inflammatory stimuli, immune cells increase the biosynthesis of membranes and organelles (for phagocytosis and motility), post-translational and epigenetic modifications (for example, supply of acetate and lactate for histone modification), free radical production, and the increased production of effector cytokines and chemokines. Mitochondria are the central organelles of metabolic reactions and regulation, and the main source of ATP and pathways for biosynthesis of macromolecules. In addition to being a central metabolism hub, they play a critical part in regulated cell death, reactive oxygen species signaling, calcium homeostasis and cell differentiation potential. Systemic, environmental, nutritional, microbiome-related and tissue-derived cues shape mitochondria through fission/fusion and mitochondrial quality control, which orchestrates how mitochondria will ultimately function and their energetic and metabolic coupling. A dysfunction in any of these processes can trigger severe disease, including chronic inflammation and neurodegeneration. Thus, it is relevant to understand how mitochondria regulation is connected to adaptive metabolism and functional output in immune and tissue-resident stromal cells in a cytokine-rich inflammatory milieu, both in physiological and pathological states. In this Research Topic, authors presented new insights of the involvement between mitochondria, oxidative stress, immune response, neurological and skeletal joint disease and regulation of circadian clock. Also, novel molecular mechanisms linking mitochondria to disease, as well as mitochondria-targeted therapies are covered by the authors.

Paul et al. assessed whether the neutrophil-to-lymphocyte ratio (NRL) is a good biomarker to assess the immune status of astronauts. They found that astronauts had higher granulocyte-to-lymphocyte (GLR) and *in vivo* and *in vitro* experiments performed in microgravity simulation revealed that microgravity increased NRL in rodents, as well imbalanced the redox processes and elevated the myeloperoxidase expression. They showed that antioxidant therapy (N-acetyl cysteine) ameliorated these effects. Also, mCAT (mitochondrial catalase) transgenic mice had reduced oxidative stress response compared to wild type. To authors, limiting ROS-drive inflammation is thus important to keep homeostatic immunity during long-term missions in space.

Wolff et al. explored the link between the circadian clock, cellular metabolism and the immune metabolic function of microglia. They reported that stimulation of microglia with SR9011, agonist of nuclear receptor Rev-erbα (involved in molecular clock and cell metabolism), disturbed the expression of metabolic and clock-related genes, decreased phagocytic activity and impaired mitochondrial respiration and ATP production. Their study provides new insights in intrinsic clock and immunometabolism of microglia. In line with this, Wang et al. also investigated the relationship between circadian clock and microglial immunometabolism. Their data revealed dysregulated expression of inflammatory and metabolic associated genes in the microglia cells of Bmal1^-/-^ mice. Bmal1 is the core transcript factor that regulates the circadian clock. Wang et al.’s data suggest that Bmal1 is a key regulator of microglial immune response and cellular metabolism.

The review of Fairley et al. summarized recent data regarding mitochondrial regulation of the microglial immunometabolism in Alzheimer’s disease (AD). They described the coordination of mitochondria in the microglial innate immunity along with nutritional, genetic and aging factors of Alzheimer’s disease. Also, they reviewed how microglial metabolism reprogramming with exercise, ketone body, mTOR and TSPO targeted-therapeutics can ameliorate AD. de Oliveira et al. discussed the importance of mitochondrial dynamics, such as mitophagy, ER-mitochondria communication and production of reactive oxygen species during neuroinflammation. They described the correlation of these mitochondrial dynamics with amelioration or worsening of central nervous system disease such as Parkinson’s and Alzheimer’s disease.

Wu et al. investigated the role of mitochondria, NLRP3 inflammasome and mitophagy in chronic intermittent hypoxia (CHI)-elicited neuroinflammation. Their *in vitro* and *in vivo* data revealed a damaging relationship of NLRP3, Parkin-dependent mitophagy and hypoxia. Authors suggest that NRLP3 knockout or pharmacological blockade can be a therapeutic strategy for CHI-elicited neuroinflammation, such as obstructive sleep apnea. Bu et al. investigated if dysfunction of mitophagy is associated with innate antiviral immunity. They described that Parkin is a negative regulator of innate immunity by facilitating degradation of RIG-I and MDA5 through K48-linked polyubiquitination of RIG-I and MDA5. Parkin is pointed by the authors as a potential therapeutic target for the control of viral infection.

In line with viral infection, Seo et al. indicated that the post-translational modification O-GlcNAcylation of the mitochondrial antiviral signaling proteins (MAVS) is important to regulate the host defense against RNA viruses. They performed experiments that revealed a heavily enriched region of O-GlcNAcylated serine in MAVS and that this modification disrupted MAVS aggregation, thus preventing MAVS-mediated activation and RLR signaling, suppressing IRF3 activation and consequently the production of type I interferon, such as IFN-β.

Teixeira et al. performed proteomic studies on myocardium tissue of patients with Chagas disease cardiomyopathy (CCC). They identified a higher frequency of dysregulated proteins involved in mitochondrial energy metabolism, cardiac remodeling and oxidative stress in CCC patients compared to patients with ischemic (IC) and idiopathic dilated cardiomyopathy (IDC). This dysregulation affected important pathways for heart function, such as fatty acid oxidation and transmembrane potential of mitochondria. Nunes et al. showed that *in vitro* stimulation of cardiomyocytes with IFN-γ and TNF-α caused increased oxidative and nitrosative stress, decreased ATP production and dependency of fatty acid oxidation, recapitulating the pathologic phenotype observed in the myocardium tissue of CCC patients. In addition, authors showed that agonists of the mitochondrial protective molecules AMPK, SIRT1 and NRF2 can ameliorate mitochondrial function of cytokine-treated cardiomyocytes. These results are relevant for several cardiac conditions where IFN-γ plays a role, like myocardial aging, myocardial infarction and anthracycline cancer chemotherapy-associated cardiopathy.

Silwal et al. discussed the double-edged behavior of mitochondrial superoxide in host defense and inflammation during infection. They pointed out that despite controlled mtROS production being essential for an efficient immune response, uncontrolled production can lead to mitochondrial damage and disease. They described the host’s mechanisms that can ameliorate mtROS generation and also how pathogens can modulate mtROS production for their own benefit. Choudhuri et al. reviewed how mitochondria health and dysfunction can influence macrophages immune response. They discussed, for example, how mitochondrial dynamics and energetics participate in the macrophage response to pathogens, the metabolic switch from oxidative phosphorylation to glycolysis, metabolic regulation and apoptosis. Like previous authors, they suggest that therapeutic strategies targeting mitochondria might be useful to control pathogenic effects of intracellular pathogens.

Yi et al. suggested that the key glycolysis enzyme Pyruvate kinase M2 (PKM2) can be a therapeutic target to treat sepsis and other inflammatory diseases. They showed that a PKM2 small-molecule agonist TEPP-46 can enhance macrophage endotoxin tolerance, increase tolerance to LPS, lethal endotoxemia and sepsis in mice. Also, TEPP-46 enhanced mitochondrial biogenesis through the key regulator PGC-1α.

Early et al. reviewed the crosstalk of mitochondria and skeletal joint diseases, such as rheumatoid arthritis and osteoarthritis. They described the central involvement of mitochondria in mechanical tissue damage, fluid-flow in bone and cartilage cells and hydrostatic pressure.

Finally, Chelombitko et al. reviewed the roles of mitochondria in the activation of mast cells through FcϵRI (Fc epsilon RI receptor). They compiled data reporting that alterations in mitochondrial membrane potential, calcium influx and reactive oxygen species have a fundamental role in the FcϵRI-dependent mast cells activation. Also, it is discussed that the PDH complex and activation of the transcription factors STAT3 and MITF are direct modulators of the mitochondrial activity of mast cells.

In summary, the articles in this Research Topic provided an outlook at the intricacies of mitochondrial and metabolic involvement in key antimicrobial and inflammatory pathways and diseases and paved the way for mitochondrial processes as therapeutic targets in infectious and inflammatory diseases.

## Author Contributions

All authors listed have made a substantial, direct, and intellectual contribution to the work and approved it for publication.

## Funding

This work was supported by the Institut National de la Santé et de la Recherche Médicale (INSERM); the Aix-Marseille University (grant number: AMIDEX “International_2018” MITOMUTCHAGAS); the French Agency for Research (Agence Nationale de la Recherche-ANR (grant numbers: “Br-Fr-Chagas”, “landscardio”); the CNPq (Brazilian Council for Scientific and Technological Development); and the FAPESP (São Paulo State Research Funding Agency Brazil (grant numbers: 2013/50302-3, 2014/50890-5, 2015/15626-8, 2019/25973-8, 2019/19435-3); the National Institutes of Health/USA (grant numbers: 2 P50 AI098461-02 and 2U19AI098461-06). This work was founded by the Inserm Cross-Cutting Project GOLD. This project has received funding from the Excellence Initiative of Aix-Marseille University - A*Midex a French “Investissements d’Avenir programme”- Institute MarMaRa AMX-19-IET-007. JPSN was a recipient of a MarMaRa fellowship.

## Conflict of Interest

The authors declare that the research was conducted in the absence of any commercial or financial relationships that could be construed as a potential conflict of interest.

## Publisher’s Note

All claims expressed in this article are solely those of the authors and do not necessarily represent those of their affiliated organizations, or those of the publisher, the editors and the reviewers. Any product that may be evaluated in this article, or claim that may be made by its manufacturer, is not guaranteed or endorsed by the publisher.

